# Large Grafting Void Resembling a Surgical Ciliated Cyst following Maxillary Sinus Augmentation. Four Case Reports with Histological Observation

**DOI:** 10.3390/medicina58091300

**Published:** 2022-09-18

**Authors:** Won-Bae Park, Meghan Pandya, Ji-Young Han, Philip Kang

**Affiliations:** 1 Department of Periodontology, School of Dentistry, Kyung Hee University; Seoul 02447, Korea; 2 Private Practice in Periodontics and Implant Dentistry, Seoul 02771, Korea; 3 Division of Periodontics, Section of Oral and Diagnostic Sciences, Columbia University College of Dental Medicine, #PH7E-110, 630 W. 168 St., New York, NY 10032, USA; 4 Department of Periodontology, Division of Dentistry, College of Medicine, Hanyang University, 222-1 Wangsimni-ro, Seongdong-gu, Seoul 04763, Korea

**Keywords:** complication, grafting void, maxillary sinus augmentation, postoperative maxillary cyst, surgical ciliated cyst

## Abstract

The cause and pathogenicity of grafting voids following lateral maxillary sinus augmentation (MSA) have not yet been elucidated. The first purpose of this case series is to introduce an unusually large grafting void that radiologically resembles a surgical ciliated cyst (SCC) at the sinus augmented site; the second is to observe the histological findings of these grafting voids. In four patients, MSA was performed using the lateral window technique. An unusually large grafting void appeared on cone-beam-computed tomography (CBCT) taken one week after surgery and except for one patient, there were no clinical symptoms. On CBCT taken six months after surgery, the grafting voids were slightly smaller in size but showed radiographic findings similar to those of SCC. During uncovering, grafting voids were removed through the lateral window site. Histologically, the grafting void was empty or filled with dense connective tissue, and no ciliated columnar epithelium or inflammatory cells were observed. Within the limitations of this case series, the large grafting voids generated after MSA was not converted to SCCs. Rather, they remained scar tissue, which could infringe the sinus bone graft and affect the apical bone support of the implant.

## 1. Introduction

Lateral sinus floor elevation is a procedure with excellent clinical predictability that enables implant placement in the severely pneumatized sinus [1,2]. Although the long-term implant survival rate with a lateral sinus floor elevation is over 90%, it is a technique-sensitive procedure due to complications that can occur during or after surgery [3]. The most common postoperative complication is maxillary sinus membrane perforation. Secondary complications such as bone graft particle leakage, sinus membrane thickening, sinus graft infection, postoperative maxillary sinusitis, and postoperative maxillary cyst have also been reported [4,5,6,7,8,9,10,11].

Among the peculiar phenomena that occur after maxillary sinus augmentation (MSA) is the appearance of voids in the bone graft. There have been reports of its clinical significance by several authors [2,7,12]. Most of them do not affect the bone graft in the maxillary sinus and are not known to have an effect on implant survival [2,12]. The exact timing and cause of the grafting void are not yet known. Air entrapment, inadequate condensation of bone graft material, and anaerobic infection were suggested as causes [2,7]. Grafting voids developed after MSA decreased in size and volume during the healing and remodelling of the bone graft [13]. Therefore, since the grafting void remained only as a radiological trace, it did not receive much attention for its clinical significance.

The postoperative maxillary cyst is also called the “surgical ciliated cyst” [6]. The surgical ciliated cyst (SCC) usually occurs after radical surgery such as Caldwell-Luc operation and orthognathic surgery performed on the maxillary sinus [14,15], but it has been reported to occur after MSA as well [4,16,17,18,19,20]. SCC is known to be caused by infiltration of the ciliated columnar epithelium into the surgical site [17,19,20].

To the authors’ knowledge, no grafting voids have been reported that resemble SCC and that is large enough to affect the apical bone support of the implant. The purpose of this case series is to examine the clinical, radiological, and histopathological findings of large grafting voids resembling SCC 6 months after MSA.

## 2. Case Presentations

The demographic information of the four patients who underwent the MSA procedure is recorded in Table 1. Written informed consent was obtained from all patients prior to the procedure.

### 2.1. Maxillary Sinus Floor Augmentation Using Lateral Window Approach

Amoxicillin 2 g was taken prophylactically 1 h before surgery. After achieving profound anaesthesia with two carpules of 2% Lidocaine 1:100,000 epinephrine with infiltration, a midcrestal incision and a vertical incision were made, and the mucoperiosteal flap was reflected buccally. The lateral window was formed using a round bur and the buccal bony lid was removed or pushed up while still attached to the mucosa. The mucosa was carefully elevated using a sinus elevation instrument (DASK kit, Dentium, Suwon, Korea). During this procedure, membrane perforation did not occur in any of the patients. The bone graft material used was xenograft (A-Oss, Osstem, Seoul, Korea) in one patient and synthetic bone graft (Osteon III, Genoss, Suwon, Korea) in the other three patients (Table 1.) The material was mixed and delivered into the elevated sinus cavity using a Molt curette. To prevent dead space, the bone graft substitute was condensed from the sinus floor. The lateral window area was treated by a membrane covering (Genoss, Suwon, Korea) or repositioning of the lateral window. Except for one patient, MSA and implant placement (Implantium, Dentium, Suwon, Korea) were performed simultaneously (Table 1). To obtain initial fixation, the osteotomy site was under-sized, and countersinking was not performed. The flap was closed using 4-0 Nylon (Ethilon^®^ 4.0, Ethicon, Cincinnati, OH, USA). Ciprofloxacin 500 mg (Yuhan Co. Seoul, Korea) and a nonsteroidal anti-inflammatory drug were prescribed for 7–10 days. Gargling of 0.12% chlorhexidine solution (Hexamedine, Bukwang Pharmaceutical, Seoul, Korea) twice a day was recommended, and the patient was informed to avoid excessive nose blowing and inhalation. Sutures were removed after 7–10 days.

### 2.2. Case 1

**Figure 1:**
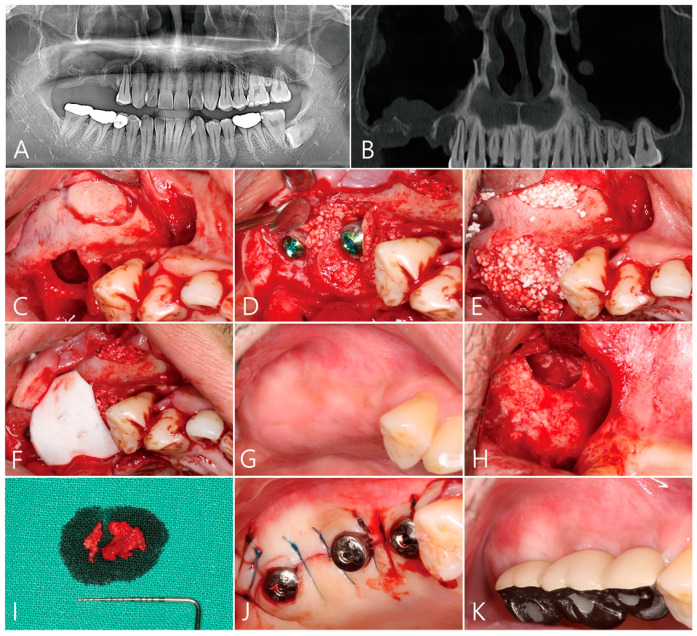
Case 1. (**A**) Preoperative panoramic radiography. A severe defect is present in the maxillary right posterior extraction socket, and the height of the residual bone is low; (**B**) panoramic image of CBCT taken preoperatively. A compromised extraction socket is observed in the right maxillary sinus, and the maxillary sinus membrane is thickened; (**C**) after the flap was reflected, the granulation tissue in the extraction socket was thoroughly removed. The buccal bone defect was severe. A lateral bone window was prepared for sinus floor elevation; (**D**) the maxillary sinus bone graft using synthetic bone graft substitute was performed and two implants were installed; (**E**) the peri-implant defect was additionally filled with a bone graft substitute; (**F**) resorbable collagen membrane was used to cover the grafting material and the flap was closed; (**G**) clinical findings 6 months after surgery. Healing was uneventful; (**H**) After the flap was reflected, the graft void was removed; (**I**) the removed specimen which was not clearly separated from the surrounding tissue; (**J**) after insertion of the healing abutment, the flap was closed; (**K**) Clinical appearance after delivery of the prosthesis.

Patient 1 is a 49-year-old male smoker with no systemic diseases that would affect the operation. On the right maxilla, posterior teeth were extracted due to periodontal disease (Table 1). Some sinus floor defects and thickening of the maxillary sinus membrane were observed on the panoramic radiograph and on the CBCT which were taken two months after the extractions were completed (Figure 1A,B).

After the flap was reflected, the granulation tissue in the extraction socket was thoroughly removed. The defect in the buccal root area was severe. A lateral bone window was prepared for sinus floor elevation (Table 1, Figure 1C). The maxillary sinus bone graft was performed using Osteon III (Genoss, Suwon, Korea) and three implants (Ø 4.3 × 10 mm Implantium, Dentium, Suwon, Korea) were placed (Figure 1D). The residual peri-implant defect was additionally filled with bone graft material (Figure 1E). The bone graft site was covered with a resorbable collagen membrane (Genoss, Suwon, Korea) and the flap was closed (Figure 1F). Postoperative clinical symptoms were not significantly different from those of general maxillary sinus surgery patients. Clinically, 6 months after the operation, there was no wound exposure and the healing was uneventful (Figure 1G). The uncovering procedure was performed under local anaesthesia. To remove the large grafting void, the mucoperiosteal flap was sufficiently reflected buccally, and then the graft void was removed using the #15 Bard-Parker blade and periodontal curettes (Figure 1H). The specimen did not separate cleanly from the surrounding tissues (Figure 1H). The grafting void and surrounding tissues were fibrotic (Figure 1I). The removed specimens were fixed in 10% formalin for biopsy. After debridement around the grafting void, no additional bone grafting was performed. This is because the shape of the defect contained a 3-walled osseous housing. After insertion of the healing abutment, the flap was closed (Figure 1J). After 2 months, the prosthesis was delivered (Figure 1K).

**Figure 2:**
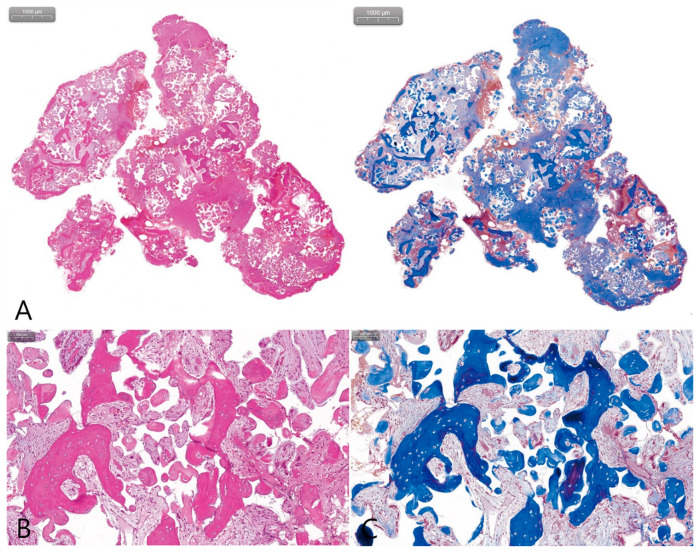
(**A**) Histopathological findings of the removed specimen (H-E stain, M-T stain); (**B**) there were many empty spaces between tissues, and no ciliary clumnar epithelium and inflammatory cells were found; loose connective tissue and woven bone were observed.

Histologically, the specimen was filled with bone graft particles and woven bone in the fibroblastic stroma, and no ciliated columnar epithelium was observed (Figure 2A). At a high magnification of the H-E stain, fibrotic stroma was filled between new bones and there were no inflammatory cells (Figure 2B). In the M-T stain at high magnification, there was no matured bone and only woven bone was observed (Figure 2B). Histologically, the grafting void, in this case, was neither an SCC nor a sign of sinus graft infection.

**Figure 3:**
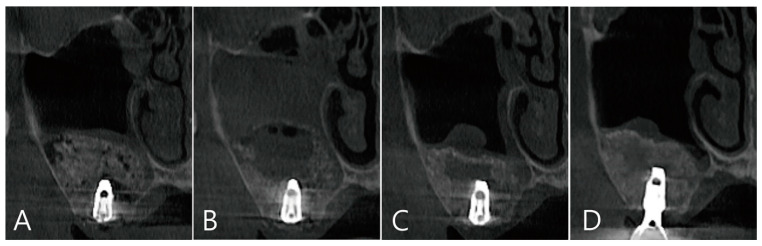
(**A**) Coronal image of the CBCT taken immediately after surgery. There was no perforation of the maxillary sinus membrane and no leakage of the bone graft substitute; (**B**) coronal image of the CBCT taken one week after surgery. A severely enlarged grafting void was observed above the implant apex, and the ostium was obstructed due to severe membrane thickening; (**C**) coronal image of the CBCT taken 6 months after surgery. Although the grafting void was reduced in size, it appears similar to the SCC; (**D**) the radiopacity of the removed grafting void site was increased in the CBCT images taken 6 months after the prosthesis was delivered.

CBCT was taken immediately after MSA, one week after surgery, and before the uncovering procedure (Figure 3A–C). On the coronal image of the CBCT taken immediately after surgery, it was confirmed that MSA and implant placement were performed at the same time, and no dislocation of the bone graft particles was observed (Figure 3A). On the coronal image of the CBCT taken one week after surgery, severe grafting voids occurred on the upper part of the implant apex, and the maxillary sinus membrane was also thickened severely (Figure 3B). On the CBCT taken just before the uncovering procedure, the grafting void decreased in size, but a radiolucent image resembling SCC was observed. The thickened maxillary sinus mucosa was also reduced (Figure 3C). The grafting void was removed and a prosthesis was delivered. On the CBCT taken 6 months after the prosthesis was delivery, the radiopacity of the removed grafting void site was increased (Figure 3D).

### 2.3. Case 2

**Figure 4:**
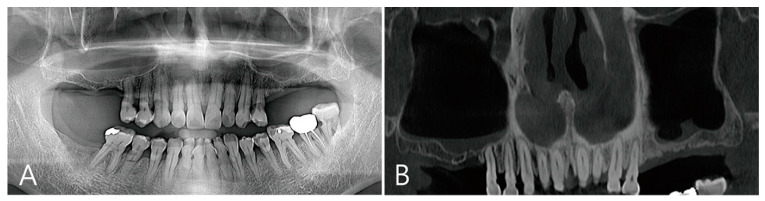
Case 2. (**A**) On preoperative panoramic radiography, severely atrophied residual ridges were observed in the missing area of the left and right maxillary molars, and the maxillary sinuses were pneumatized; (**B**) a panoramic image of the preoperative CBCT showed slight thickening of the sinus membrane on the sinus floor.

**Figure 5:**
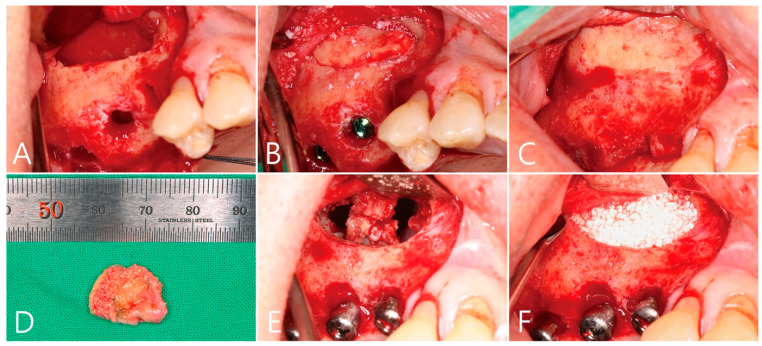
(**A**) After flap reflection, the lateral bone window was removed and sinus floor elevation was performed. There was no perforation of the maxillary sinus mucosa. Three osteotomy sites were perparated for implant placement; (**B**) the maxillary sinus was filled with xenograft material and the removed lateral window bone was repositioned. An implant was also placed. The flap was closed without barrier membrane covering; (**C**) the repositioned lateral bone window after 6 months of operation was well integrated with the adjacent native bone; (**D)** the previously repositioned lateral window was separated again to access the remaining large grafting void. The shiny appearance of the inner aspect of the removed specimen is the mucous membrane in contact with the grafting void, and to the left is the lateral window bone; (**E**) after the grafting void was removed, some perforation of the maxillary sinus mucosa was observed in the maxillary sinus bone graft; (**F**) after repair with resorbable collagen membrane, synthetic bone graft substitute was filled into the void.

Patient 2 is a 72-year-old female non-smoker with hypertension who had been taking antihypertensive drugs for a long time (Table 1). This patient visited the clinic for implant placement of the maxillary left and right posterior teeth. Preoperative panoramic radiography and CBCT were taken. There were also severely atrophied residual ridges and pneumatized maxillary sinuses of the edentulous ridges in the maxillary posterior regions (Figure 4A). There was no thickening of the sinus membrane as a panoramic image of the preoperative CBCT (Figure 4B). A lateral bone window was prepared using the lateral window technique. There was no perforation of the maxillary sinus mucosa during the sinus floor elevation process. Three osteotomy sites were prepared for implant placement (Figure 5A). The elevated sinus cavity was filled with xenograft (A-Oss, Osstem, Seoul, Korea) and the removed lateral window bone was repositioned. Three implants (Ø 4.3 × 10, Implantium, Dentium, Suwon, Korea) were placed (Table 1). The flap was closed without a barrier membrane covering (Figure 5B). There were no complications during the healing process and no adverse events. Recovery was performed 6 months after surgery. The repositioned lateral window site was well fused to the surrounding native bone (Figure 5C). To remove the large grafting void, the repositioned lateral window also had to be removed. The removed specimen (1.5 × 1.2 cm) appeared shiny on the inner surface and was lined with soft tissue (Figure 5D). In the area where the grafting void was removed, some of the maxillary sinus mucosae were also removed and perforation occurred (Figure 5E). The perforated mucosa was covered with a resorbable collagen membrane (Genoss, Suwon, Korea) and then filled with bone graft material (Osteon III, Genoss, Suwon, Korea). After the healing abutment was inserted, the flap was closed (Figure 5F).

**Figure 6:**
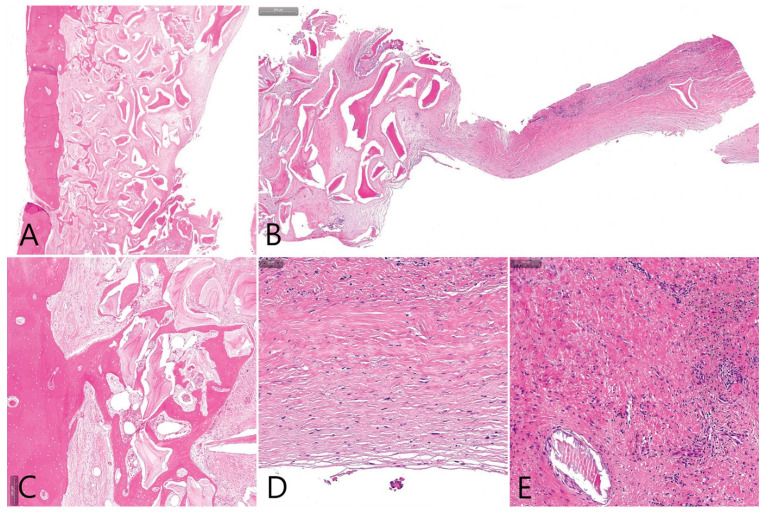
(**A**) Histopathological findings of the removed specimen (H-E stain). On the left side of the specimen is the cortical layer of the lateral bone window. To the right of the bone is the maxillary sinus bone graft. The empty space on the far right is the grafting void; (**B**) soft tissue surrounding the grafting void connected to the bone graft was observed; (**C**) new bone formation was observed in the area adjacent to the lateral window; (**D**) the soft tissue surrounding the grafting void was dense connective tissue, and no epithelial cells were observed; (**E**) a large number of fibroblasts were distributed in the dense connective tissue, and no inflammatory cells were observed.

The removed specimen was fixed in 10% formalin. Histological observation was performed after H-E stain was applied. The cortical layer located on the left side of the specimen is the lateral bone window, and to the right of that is the maxillary sinus bone graft. The empty space on the right side is the grafting void. No new bone was found around the bone graft particles. (Figure 6A). Soft tissue surrounding the grafting void connected to the bone graft was observed. No bone graft particles were found in the attached soft tissue (Figure 6B). It was observed that the partially formed new bone started from the lateral window and progressed into the sinus graft (Figure 6C). The soft tissue surrounding the grafting void was dense connective tissue, and no epithelial cells were observed (Figure 6D). A large number of fibroblasts were distributed in the dense connective tissue, and no inflammatory cells were observed (Figure 6E).

**Figure 7:**
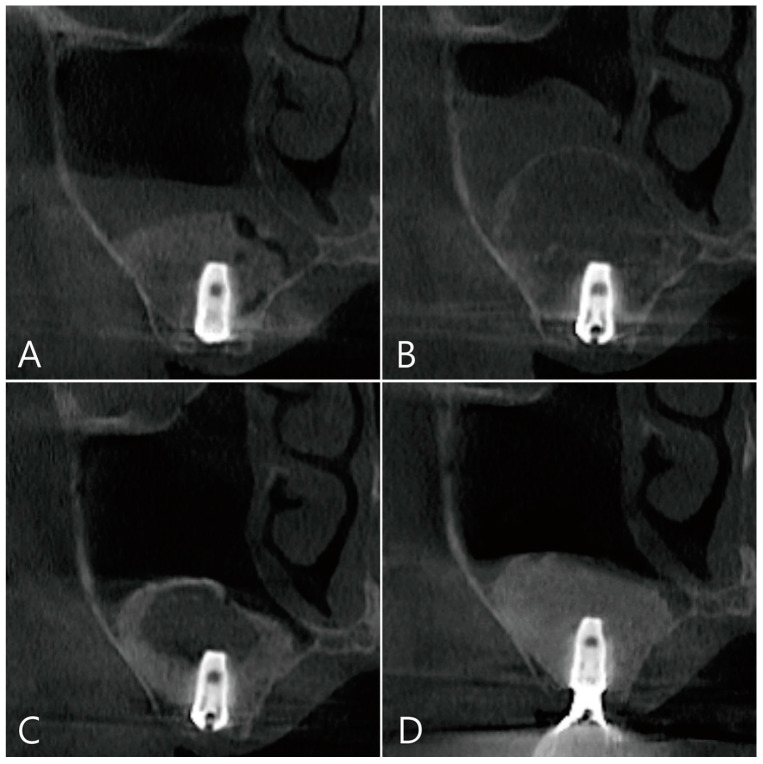
Coronal images of the CBCT were taken at multiple points during the healing process after surgery: (**A**) an image taken immediately after surgery; (**B**) an image taken one week after surgery. A large grafting void occurred over the apical half of the implant, and thickening of the maxillary sinus mucosa was also observed; (**C**) in the image 6 months after surgery, the size of the grafting void was slightly reduced. The void presents with a cystic appearance resembling SCC. The apical bony support of the implant was lost. The grafting void was removed and additional bone grafting was performed; (**D**) in the CBCT image taken 6 months after the prosthesis was delivered, it can be confirmed that the previous grafting void was replaced with a bone graft substitute.

The coronal images of CBCT taken during the healing process after surgery were examined. There was no leakage of bone graft particles in the CBCT taken immediately after surgery (Figure 7A). One week after surgery, a large grafting void was developed over the apical third of the implant, and thickening of the sinus membrane was observed (Figure 7B). The shape of the grafting void after 6 months of surgery became clear and the size decreased. The sinus membrane thickening was also reduced. The void was filled with homogenous contents and showed a cystic appearance (Figure 7C). In the CBCT taken 6 months after the prosthesis was delivered, the bone graft substitute filled the grafting void well (Figure 7D).

### 2.4. Case 3

This patient was a 63-year-old male smoker who was being treated for hypertension and diabetes. The maxillary sinus bone graft was performed after the maxillary right posterior teeth were extracted due to severe periodontitis. There was no perforation of the sinus membrane during sinus floor elevation, and Osteon III (Genoss, Suwon, Korea) was used as a bone graft material (Table 1). Lateral window sites were treated by repositioning the removed lateral windows. No adverse events occurred during the healing process.

**Figure 8:**
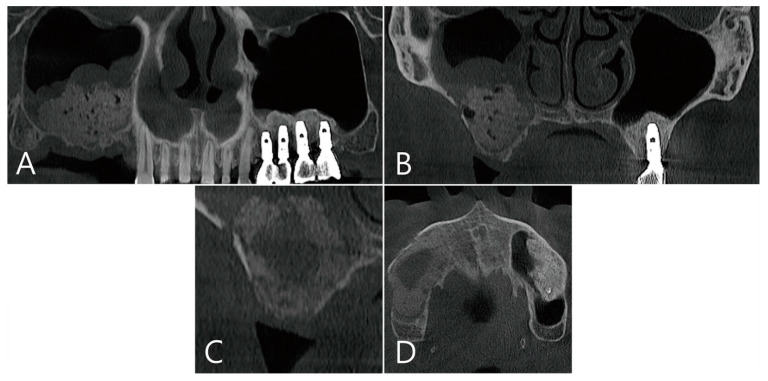
Case 3. CBCT images taken immediately after maxillary sinus bone graft: (**A**) On the panoramic image of CBCT, a thickened mucosa on the sinus floor was observed above the bone graft; (**B**) Coronal image of the CBCT. (**C**,**D**) Images of the CBCT taken 6 months after surgery: (**C**) in the coronal image of the CBCT, large grafting voids with a cystic appearance were observed; (**D**) a well-defined radiolucent appearance was observed on an axial image of the CBCT.

In the CBCT image taken immediately after the maxillary sinus bone graft, a thickened sinus membrane was observed on top of the bone graft material, and there was no leakage of the bone graft material (Figure 8A,B). In the CBCT image taken 6 months after surgery, a large grafting void was observed in the bone graft area which showed a cystic appearance (Figure 8C,D).

**Figure 9:**
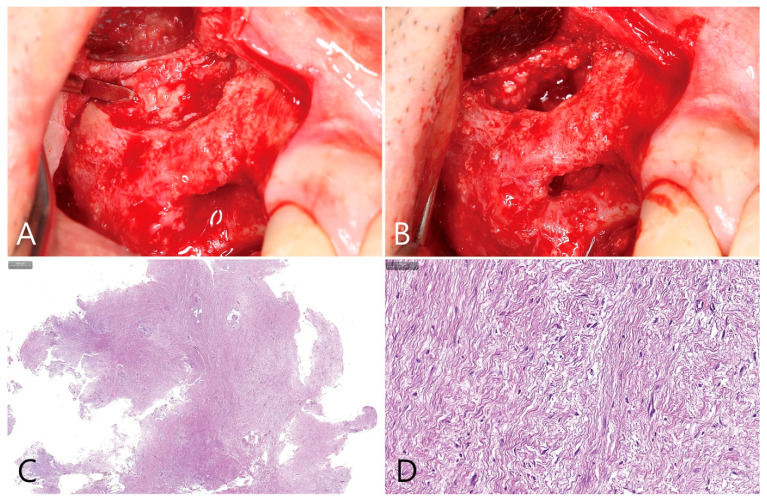
(**A**) Six months after surgery, the flap for implant placement was reflected. At this time, the grafting void was accessed through the lateral window site; (**B**) the specimen was very fibrotic and was removed using a curette. The specimen was not well separated from the surrounding regenerated bone tissue. This may compromise the apical bony support for future implant placement; (**C**) the surface of the removed specimen was very irregular and no ciliated columnar epithelium was observed; (**D**) at high magnification, dense fibrotic tissue was observed and there was no infiltration of inflammatory cells.

Implant placement was performed 6 months after surgery. After removing the lateral window repositioned during lateral window technique, the grafting void in the bone graft was removed using a periodontal curettes. The specimen was very fibrotic and difficult to separate from the surrounding bony tissues (Figure 9A). The grafting void was connected to the previous tooth extraction socket. It was large enough to affect implant placement (Figure 9B). Histologically, the specimen showed a very irregular appearance, and inflammatory cells and ciliated columnar epithelium were not observed. The grafting viod was entirely filled with dense connective tissue (Figure 9C,D).

### 2.5. Case 4

**Figure 10:**
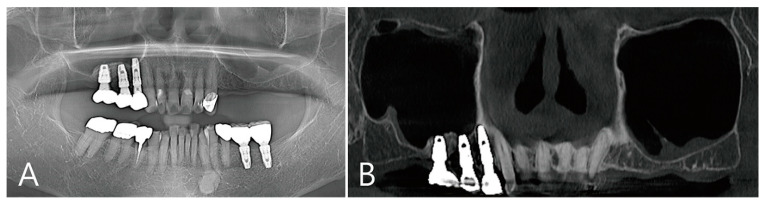
Case 4. Preoperative panoramic radiography and CBCT scan were performed: (**A**) the left maxillary sinus was severely pneumatized and had minimal residual bone height; (**B**) in the maxillary sinus, membrane thickening was confined to the sinus floor and no sinus pathology was observed in the remaining areas.

A 75-year-old female who was a non-smoker visited a private clinic to have the implant placed in the edentulous ridge of the maxillary left posterior region (Table 1). This patient was taking antihypertensive and antihyperlipidemic drugs. Preoperative panoramic radiography and CBCT scan were performed. The left maxillary sinus was severely pneumatized and had a minimal residual bone height of 2–4 mm, rendering normal implantation impossible (Figure 10A,B). In the maxillary sinus, membrane thickening was confined to the sinus floor and no sinus pathology was observed in the remaining areas (Figure 10B). Lateral MSA and simultaneous implant placement were planned on the left maxillary sinus.

**Figure 11:**
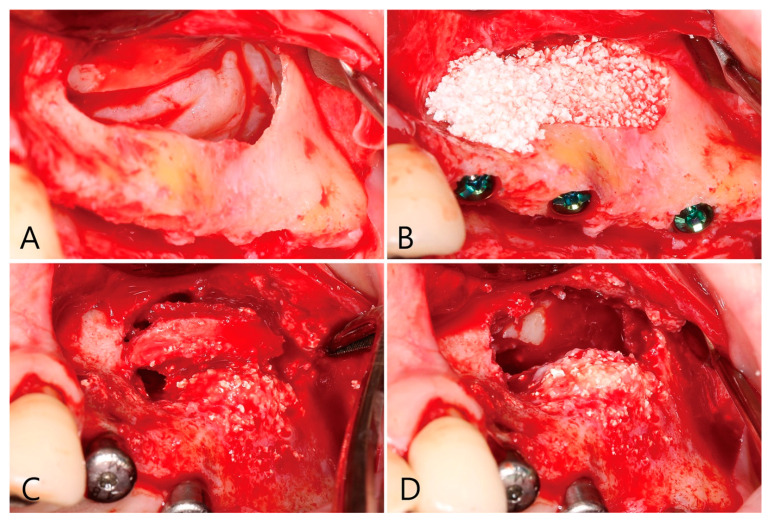
(**A**) The ovular lateral window was formed using a surgical round bur on the buccal bone. The Schneiderian membrane was detached from the sinus floor and elevated. No perforation of the Schneiderian membrane occurred; (**B**) biphasic calcium phosphate was mixed with physiological saline and placed in the maxillary sinus. Three implants were simultaneously placed; (**C**) six months after the MSA, when the buccal flap was reflected, the buccal bone which was interconnected with the void was removed. Healing abutments were inserted into the implants; (**D**) gentle debridement and saline irrigation were performed on the removed voided site. The removed site was not refilled with a bone graft substitute.

During the sinus floor elevation, no perforation of the Schneiderian membrane occurred. The lateral bony window was pushed up and remained attached to the Schneiderian membrane (Figure 11A). Biphasic calcium phosphate (Osteon™ III, Genoss, Suwon, Korea) was selected as a bone graft substitute, the bone graft substitute was condensed toward the sinus floor. Three implants (Implantium, Dentium, Suwon, Korea) were simultaneously placed (Table 1). The lateral window site was covered with a collagen membrane (Genoss, Suwon, Korea) (Figure 11B) and the flaps were closed with tension-free closure. One week after the operation, the patient complained of severe pain. I&D was performed and antibiotics (Augmentin 625 mg, Ilsung Pharmaceuticals, Seoul, Korea), as well as an anti-inflammatory drug, were prescribed for an additional week. There were no clinical signs until the uncovering procedure. Six months after the MSA, the uncovering procedure was performed. The buccal bone, which was interconnected with the void was removed to eliminate the infection source (Figure 11C). Gentle debridement and saline irrigation were performed on the removed voided site (Figure 11D). The removed site was not refilled with a bone graft substitute due to the possibility of residual infection. Healing abutments were inserted into the implants. The flap was closed with tension-free closure.

**Figure 12:**
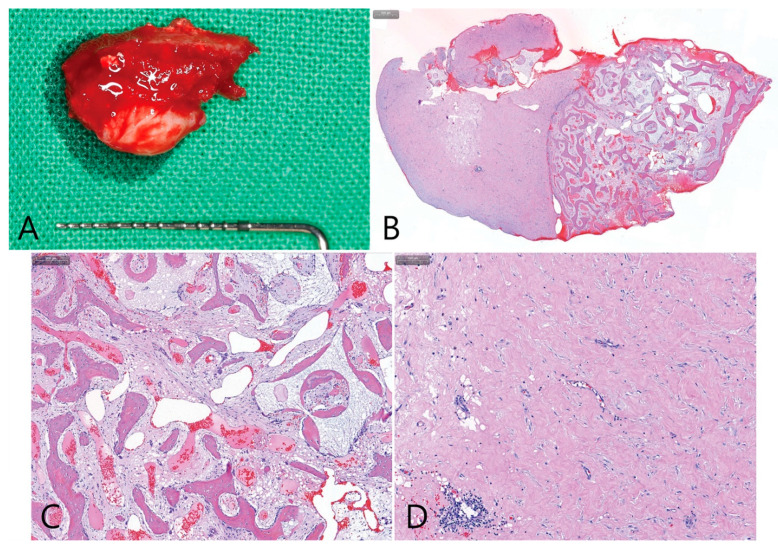
(**A**) The removed specimen was fixed in 10% formalin solution for histopathological examination; (**B**) the specimen was examined after hematoxylin and eosin (H&E) staining. Newly formed bone and void tissue were clearly bordered; (**C**) new bone formation was found around the bone graft particles, and osteoblasts, osteocytes, and osteoclasts were observed; (**D**) no new bone formation was observed in the void area. Epithelial cells containing cilia were not observed in the void tissue which consisted only of dense connective tissue.

The size of the removed specimen was 1.5 × 1.0 cm, and the soft tissue was very firm. The specimen was not separated into several parts but was removed as a single piece (Figure 12A). The specimen was examined after hematoxylin and eosin (H&E) staining. Newly formed bone and void tissue presented with a clear border (Figure 12B). New bone formation was found around the bone graft particles, and osteoblasts, osteocytes, and osteoclasts were observed (Figure 12C). No new bone formation was observed in the void area. Epithelial cells containing cilia were not observed in the void tissue which consisted only of dense connective tissue (Figure 12D).

**Figure 13:**
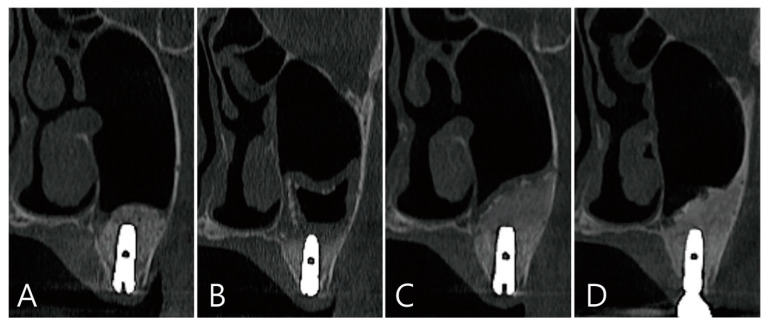
The coronal image of the CBCT scanned at the #27 implant site was examined: (**A**) in the image taken immediately after surgery, there was no leakage of bone graft particles; (**B**) however, a very large grafting void appeared in the image taken one week after surgery; (**C**) this grafting void showed a cystic appearance in the implant apex, although its size was reduced on CBCT taken after 6 months; (**D**) in CBCT taken 2 years after the grafting void was removed, the grafting void was replaced with new bone.

The coronal image of the CBCT scanned at the #27 implant site was examined. In the image taken immediately after surgery there was no leakage of bone graft particles (Figure 13A), however, a very large grafting void appeared in the image taken one week after surgery (Figure 13B). This grafting void showed a cystic appearance at the implant apex, although its size was reduced on CBCT taken after six months (Figure 13C). On the CBCT taken two years after the grafting void was removed, the grafting void was replaced with new bone (Figure 13D).

## 3. Discussions

The appearance of large grafting voids at the sinus augmented site resembled SCC and affected the apical bony support of the implant. Excision with or without additional bone graft material should be considered during the uncovering procedure.

In general, SCC is known to be caused by entrapment of the ciliated columnar epithelium when radical sinus surgery such as Caldwell-Luc operation or orthognathic surgery is performed on the maxillary sinus [14,15]. In addition, SCCs occur rarely after maxillary sinus bone graft, and the onset period is usually reported to be between 2–10 years [4,17,18,19,20]. However, postoperative maxillary cysts detected six or nine months after surgery have also been reported [16,20]. The presented cases show grafting voids which radiologically resemble SCCs six months after surgery. If a CBCT was not taken one week after MSA or if a biopsy was not performed at the time of uncovering, the large grafting void would have been misdiagnosed as an SCC.

The common consensus is that grafting voids generated after MSA do not affect maxillary sinus bone graft and implant survival rate [2,12]. Therefore, grafting voids were not recognized as early complications of MSA. The exact mechanism for void formation is also unknown. Incomplete bone formation [21], anaerobic infection [7], and air entrapment [2] were suggested to be the causes. In addition, grafting voids are sometimes found in the early stages of sinus graft infection, so they are also presumed to be bacterial infections with clinical manifestations. However, in the presented cases, except for case 4, clinical symptoms did not appear, and infiltration of inflammatory cells was not detected in the specimens, which is different from a sinus graft infection. The clinical symptoms that occurred in case 4 were due to the sudden expansion of a large grafting void without sinus membrane thickening. The pain was relieved by air ventilation through I&D.

All the voids occurred one week after the maxillary sinus bone graft, and the voids appear to have occurred in the early stages of the maxillary sinus bone graft. Nevertheless, these cases were very large in size, which affected the apical bony support of the implant and resulted in a decrease in the total graft volume. Moreover, since radiologically the lesion appears to look like SCC, it was decided to remove it during the uncovering procedure as leaving SCC without treatment would lead to a more aggravated condition in the future. Park et al. reported a case of postoperative maxillary cyst involving the nasal cavity due to sudden enlargement of a small radiolucent lesion in the maxillary sinus bone graft [22]. In the present cases, the large grafting void was removed, an additional bone graft was performed, and the area with a rather small defect was left without graft.

There are few reports of histopathological findings of grafting voids. Kang et al. reported that grafting voids were found in CBCTs taken 6 months after maxillary sinus bone graft using synthetic bone graft/rh BMP-2, and histologically, new bone and fatty marrow-like tissue without ciliated columnar epithelium were observed [13]. However, Kang et al. examined the specimen obtained through the osteotomy site for implant placement [13], therefore only a limited portion could be observed compared to the specimen obtained through the lateral window site in present cases. In the four specimens presented in these reports, ciliated epithelium of the SCC was not observed and there were no inflammatory cells. One out of four cases was empty and three were filled with dense connective tissue. This showed that the appearance of grafting voids was independent of the influx of ciliated cells of the Schneiderian membrane. The present case reports show that the large grafting void appears to be SCC radiologically, but histologically, it is not related to the appearance of SCC and sinus graft infection. However, large voids reduce the volume of the sinus bone graft and infringe upon the apical bony support of the implant. Another possibility of void formation is the invasion of inflammatory cells or fibroblasts which can form granulation tissues near the superior border of the graft material that is in close contact with the inflamed Schneiderian membrane, utilization of resorbable membranes for better cell occlusion can also be considered to prevent epithelial migration into the grafts.

This case series has limitations in reaching a definite conclusion due to the limitation of the number of patients, and it is thought that a controlled study involving a large sample size and many clinicians will be required in the future.

## 4. Conclusions

Within the limitations of the present case reports, if the grafting void developed after MSA is large and resembles the SCC, it should be considered one of the early complications of MSA regardless of clinical symptoms.

## Figures and Tables

**Table 1:** Demographic characteristics of patients and information on implants, bone graft, sinus perforation, and follow-up.

Case	Age/Sex	Smoking	Implant Sites	Implant Size (mm)	Bone Graft	Sinus Perforation	Follow-Up (Months)
1	49/M	Yes	#15 #16 #17	4.3 × 10 4.3 × 10 4.3 × 10	Synthetic	No	6
2	72/F	No	#15 #16 #17	4.3 × 10 4.3 × 10 4.3 × 10	Xenograft	No	6
3	63/M	Yes	Delayed placement (right maxilla)		Synthetic	N0	6
4	75/F	No	#24 #25 #27	3.8 × 12 3.8 × 12 3.8 ×12	Synthetic	No	24

## Data Availability

Not applicable.

## Abbreviations

CBCT	Cone-beam computed tomography
H&E	Hematoxylin and eosin
I&D	Incision and drainage
MSA	Maxillary sinus augmentation
rh BMP	Recombinant human bone morphogenetic protein
SCC	Surgical ciliated cyst
